# *Rhodiola rosea* polysaccharides promote the proliferation of bone marrow haematopoietic progenitor cells and stromal cells in mice with aplastic anaemia

**DOI:** 10.1080/13880209.2022.2083187

**Published:** 2022-06-12

**Authors:** Jing Li, Yongfeng Chen

**Affiliations:** a Department of Histology and Embryology, North Sichuan Medical College, Nanchong, Sichuan, China; b Department of Basic Medical Sciences, Medical College of Taizhou University, Taizhou, China

**Keywords:** *Rhodiola rosea* L., haematopoietic progenitor cells, bone marrow stromal cells, mice with aplastic anaemia, haematopoiesis regulation

## Abstract

**Context:**

The effects of *Rhodiola rosea* L. (Crassulaceae) polysaccharides (RRPs) on haematopoiesis are poorly understood.

**Objective:**

To determine the effects of RRPs on haematopoiesis in mice with aplastic anaemia.

**Materials and methods:**

Aplastic anaemia was induced in Kunming mice by ^60^Coγ (2.0 Gy) irradiation and cyclophosphamide administration (50 mg/kg/day for 3 consecutive days; intraperitoneal injection). The *in vivo* effects of RRPs (10, 20, and 40 mg/kg; intraperitoneal injection) on haematopoiesis were analyzed using peripheral blood tests, histopathological examination of haematopoietic tissues, culture of haematopoietic progenitors and bone marrow stromal cells (BMSCs), and Western blotting of Fas and Fas ligand (FasL). The *in vitro* effects of RRPs on bone-marrow haematopoietic progenitors and BMSCs were also evaluated.

**Results:**

Compared to anaemic controls, high-dose RRPs (40 mg/kg) significantly increased red blood cells (8.21 ± 0.57835 versus 6.13 ± 1.34623 × 10^12^/L), white blood cells (5.11 ± 1.6141 versus l.54 ± 1.1539 × 10^9^/L), and BMSCs (10.33 ± 1.5542 versus 5.87 ± 3.1567 × 10^12^/L) in mice with aplastic anaemia (all *p* < 0.01). High-dose RRPs significantly increased the formation of colony-forming unit-granulocyte macrophage (CFU-GM), burst-forming unit-erythroid (BFU-E), and colony-forming unit-erythroid (CFU-E; *p* < 0.01). Fas and FasL protein expression in BMSCs decreased after RRPs administration. Especially at the high dose, RRPs (150 μg/mL) significantly promoted *in vitro* CFUs-E, BFUs-E, and CFUs-GM formation. RRPs (150–300 μg/mL) also promoted BMSC proliferation.

**Discussion and conclusions:**

RRPs helped to promote haematopoietic recovery in mice with aplastic anaemia, facilitating haematopoietic tissue recovery. This study indicated some mechanisms of the haematopoietic regulatory effects of RRPs. Our findings provide a laboratory basis for clinical research on RRPs.

## Introduction

*Rhodiola rosea* L. (Crassulaceae) is a perennial herb or subshrub (i.e., dwarf shrub) with a long history of being used as a dietary supplement and a traditional medicine in China, Russia, and other countries. In China, *R. rosea* is widely used to eliminate fatigue, improve physical activity, and alleviate altitude sickness in high-altitude areas (Petkov et al. 1986). Recent studies have identified more than 150 biologically active compounds in *R. rosea* (Shikov et al. 2020). Pharmacological research has revealed that *R. rosea* preparations exhibit an adaptogenic effect (Panossian et al. 2010; Panossian 2017). In addition, *R. rosea* has been shown to have anti-fatigue, anti-cold, anti-inflammatory, anti-radiation, anti-stress, and antidepressant (Amsterdam and Panossian 2016; Li et al. 2017; Tao et al. 2019) properties; it can also provide resistance to hypoxia, inhibit tumours, and control blood glucose levels (Shi et al. 2012; Lee et al. 2013; Leung et al. 2013; Zhang et al. 2013). In addition, extracts of *R. rosea* have certain positive effects on haematopoietic regulation (Udintsev and Schakhov 1991; Qian et al. 2011). Thus far, most pharmacological studies on *R. rosea* have focussed on its alcohol-soluble ingredients such as salidroside, rhodosin, and *p-*tyrosol (Hu et al. 2010; Li et al. 2011; Xie and Zhu 2012).

*Rhodiola rosea* polysaccharides (RRPs) are one of the main water-soluble ingredients of *R. rosea*, and primarily include glucose, galactose, mannose, rhamnose, and arabinose. Recent studies have demonstrated that RRPs are abundant in *R. rosea* and have special physiological activities and pharmacological effects.

Studies have shown that RRPs have significant hypoglycaemic effects (Chem et al. 1996; Ding 2011), antioxidative activities, and hepatoprotective effects, and could prevent and treat liver damage induced by toxic chemicals. The antioxidative activity of RRPs is probably mediated through an increase in serum superoxide dismutase and glutathione peroxidase levels along with a decrease in serum reactive oxygen species and malondialdehyde levels (Nan et al. 2003; Huang et al. 2012; Song et al. 2015; Xu et al. 2018). *In vitro* and *in vivo* experiments have shown that RRPs have a potent tumour-growth inhibition effect on Sarcoma 180 cells. The underlying mechanism includes increase in the production of serum interleukin-2, tumour necrosis factor-α, and interferon-γ, which elevates the ratio of CD4^+^/CD8^+^ T lymphocytes in tumour-bearing mice (Cai et al. 2012). *Rhodiola rosea* has also been demonstrated to have an anti-radiation effect and could promote the recovery of haematopoietic function (Gao et al. 1994; Huang and Jiang 2003; Li and Luo 2012; Lei et al. 2014). Genotoxicity tests, including the Ames test, mouse BMC micronucleus test, and mouse sperm abnormality test, demonstrated that RRPs are safe, with no toxicity or mutagenicity (Liu et al. 2022).

However, systematic research on RRPs, particularly from the perspective of haematopoietic regulation, is rare. Therefore, the present study aimed to determine the influence of RRPs on bone-marrow haematopoietic function in a mouse model of aplastic anaemia. The purpose of this study was to enhance the exploitation and utilization of *R. rosea*.

## Materials and methods

### Experimental materials

RRPs were provided by Mingbo Company (Chengdu, China). Cyclophosphamide injections were purchased from Shanghai Hualian Pharmaceutical Co. Ltd. (Shanghai, China). Iscove’s modified Dulbecco’s medium (IMDM) substratum, granulocyte-macrophage colony-stimulating factor, thrombopoietin, and erythropoietin were obtained from Jingmei Co. (Chengdu, China). The following equipment was used: a fully automatic blood-cell analyzer (Hema-Screen18, Hospitex Diagnostics, Italy), a flow cytometer (Beckman Co., Brea, CA), an inverted-phase contrast microscope (Chongqing Optical Instrument Factory, Chongqing, China), a CO_2_ incubator (MCD-15A, Japan), and a fully automatic enzyme-linked immunosorbent assay (ELISA) metre (Thermo Fisher Scientific, Waltham, MA). The radiation source (^60^Coγ) was procured from the Sichuan Academy of Agricultural Sciences (Chengdu, China).

### Animals

A total of 105 healthy Kunming male mice (weight, 18–22 g each; age, 6–8 weeks) were obtained from the Experimental Animal Centre, Chengdu University of Traditional Chinese Medicine (Certification No. CSDZG-7). The study was approved by the Animal Ethics Committee of Chengdu University of Traditional Chinese Medicine, China. All procedures performed in studies involving animals were in accordance with the ethical standards of the institution or practice at which the studies were conducted.

### Preparation of RRP solution

RRPs were diluted in normal saline to obtain solutions with concentrations of 1, 2, and 4 mg/mL. These solutions were stored at 4 °C until analysis.

### Establishment of mouse model of aplastic anaemia

The mice were randomly divided into five groups as follows: normal-control group (*n* = 20), aplastic anaemia control group (*n* = 25; to account for early deaths), and three RRP groups with low, medium, and high doses of RRPs (10, 20, and 40 mg/kg, respectively; *n* = 20 each). The aplastic anaemia model was established using the method described by Zhang et al. (2006). The mice in the aplastic anaemia and RRP groups were irradiated with 2.0 Gy ^60^Coγ and then intraperitoneally injected with cyclophosphamide at a dose of 50 mg/kg/day for 3 consecutive days. After the modelling, the mice in the RRP groups received daily 0.2 mL intraperitoneal injections of high-, medium-, and low-dose RRPs for 7 consecutive days. During this time, the mice in the normal- and anaemic control groups were intraperitoneally injected with the same volume of normal saline (i.e., 0.2 mL/day).

### Peripheral blood cell and bone marrow cell counts

Routine blood tests were performed 24 h after the last RRP/saline injection in each group. From each group, 10 mice were sacrificed, and their femurs harvested. The harvested bones were used to prepare BMC suspensions according to a routine method (Zhang et al. 2005), and the BMC counts were noted.

### Histopathological examination

From each group, six randomly selected mice were sacrificed. Their femurs were harvested, fixed in 4% neutral formalin, decalcified, desiccated, paraffin embedded, cut into semi-thin sections, stained with haematoxylin and eosin, and examined using light microscopy to evaluate the histopathological changes in the bone marrow.

### *In vivo* effects of RRPs on colony formation by bone-marrow haematopoietic progenitors

A portion of the BMC suspensions prepared for the cell count assessments was used for *in vitro* cultivation. The BMC suspension was diluted to a concentration of 1 × 10^5^ cells/mL, and then cultured at 37 °C under 5% CO_2_ with saturated humidity. The culture system was consistent with the method described by Chen et al. (2009). After 3 days, we counted the number of colony-forming units-erythroid (CFUs-E), and after 7 days, we counted the number of CFUs-E, burst-forming units-erythroid (BFUs-E), colony-forming units-granulocyte-macrophage (CFUs-GM), and colony-forming units-megakaryocyte (CFUs-Meg).

### Western blot analysis of Fas and Fas ligand expressions

Protein concentrations were determined using the Bradford method. The total protein extraction was conducted by adding a protein isolation solution to the BMC suspension. Samples of the protein extracts were boiled for 3 min in a water bath, and 50 μg total proteins were separated using 10% sodium dodecyl sulphate polyacrylamide gel electrophoresis and then transferred to polyvinylidene difluoride (PVDF) membranes. The membranes were blocked with 5% skim milk for 1 h and then incubated overnight with the primary antibodies at 4 °C. Next, the membranes were rinsed three times with Tris-buffered saline supplemented with Tween 20 (TBST), incubated for 1 h with the appropriate secondary antibodies (anti-Fas and anti-Fas ligand [FasL]) at room temperature, and again washed three times with TBST. Finally, Fas and FasL expressions were measured using enhanced chemiluminescence and ImageQuant LAS 4000 (GE, New York, NY, USA).

### *In vitro* effects of RRPs on colony formation by bone-marrow haematopoietic progenitors

Six anaemic mice were sacrificed, and their femurs were harvested and used to prepare BMC suspensions (concentration, 1 × 10^5^ cells/mL). The suspensions were divided into five groups with differing RRP concentrations. The basal culture medium was the same as that used to test the *in vivo* effects of RRPs, except that it was supplemented with IMDM or different concentrations of RRPs. The final RRP concentrations in the five *in vitro* groups were 0, 50, 100, 150, and 200 μg/mL. The cells were cultured for 7 days at 37 °C under 5% CO_2_ and saturated humidity, and colony formation was observed using inverted-phase contrast microscopy.

### *In vitro* effects of RRPs on the proliferation of bone marrow stromal cells

Six normal mice and six anaemic mice were sacrificed, and BMC suspensions were prepared as described above. Their concentrations were adjusted to 2 × 10^7^ cells/mL, and the suspensions were divided into 12 groups: 6 normal-RRP groups and 6 model-RRP groups. Each group contained six 200 μL samples of BMC suspensions. After 24 h of culture, non-adherent cells were eliminated. The culture fluid was then removed, and fresh culture fluid containing different concentrations of RRPs was added, to obtain final concentrations of 0, 50, 100, 150, 200, and 300 μg/mL. The proliferation of bone marrow stromal cells (BMSCs) was observed after 24, 48, and 72 h of culture at 37 °C under 5% CO_2_ with saturated humidity. BMSC proliferation was analyzed using MTT assays (Moura et al. 2012). At 4 h before the end of each culture period, most of the culture fluid was removed, and MTT was added to each well. Then, the culture plate was placed back into the incubator. After 4 h, the MTT was removed, and the cells were washed twice with phosphate-buffered saline. Next, 100 μL isopropyl alcohol was added to lyse the cells. Finally, ELISA was performed to detect the absorbance of each well at 570 nm, and cell proliferation was measured as follows:

Cell proliferation rate = (absorbance value of the experimental groups − average absorbance value of the control groups)/average absorbance value of the control groups × 100%.

### Statistical analysis

Data are presented as means and standard deviations. Analysis of variance was used to assess between-group differences, and *p* = 0.05 was deemed to indicate statistical significance.

## Results

### Effects of RRPs on peripheral blood

**Figure 1.**
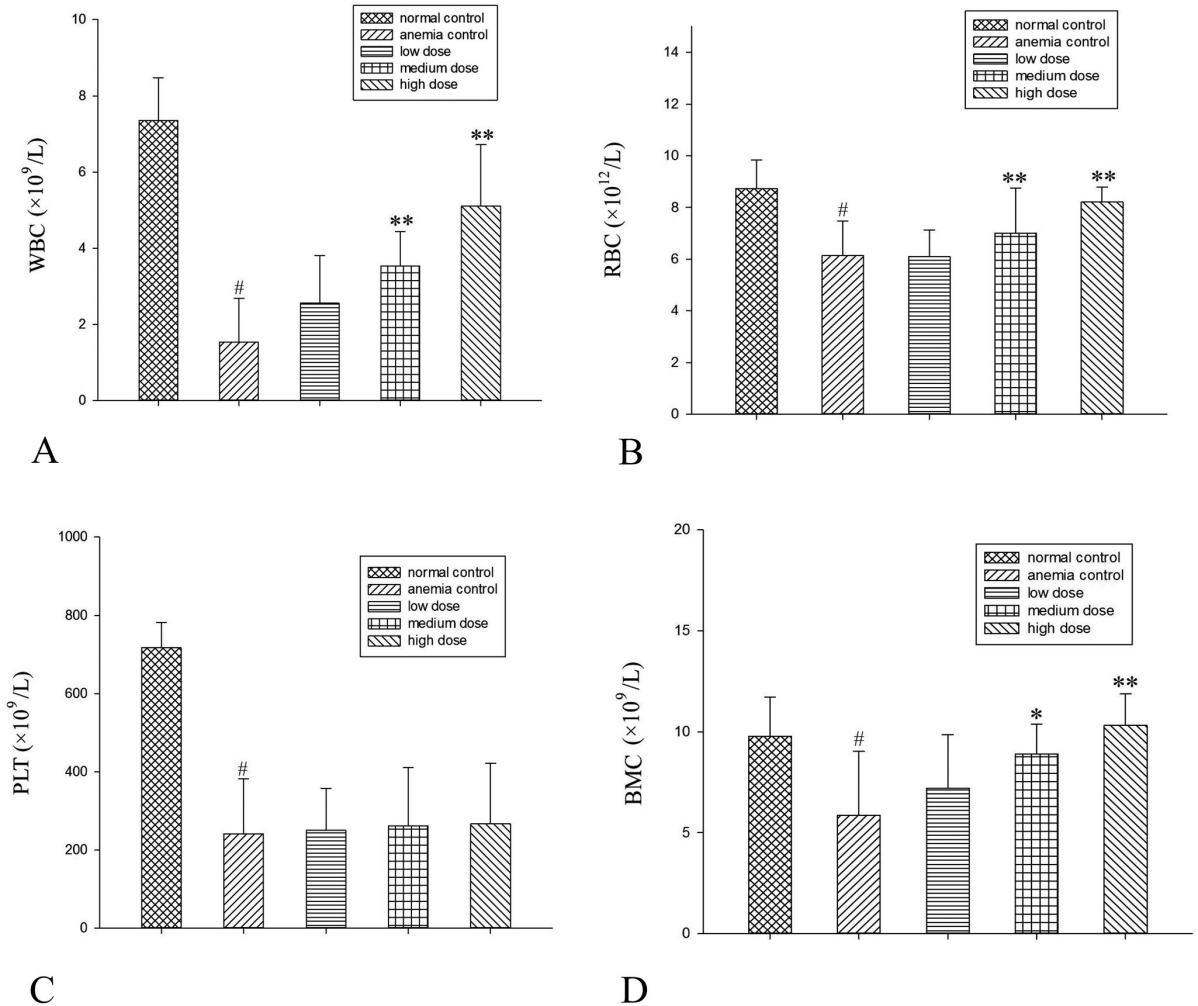
Influence of RPPs on peripheral blood and BMCs in mice with aplastic anaemia. (A) WBCs, (B) RBCs, (C) PLTs, and (D) BMCs. #*p<*0.01 versus normal controls; *p < 0.05 and **p < 0.01 versus anaemic controls.

RRPs elevated the red and white blood cell counts in a dose-dependent manner in mice with aplastic anaemia, but failed to elevate the platelet count. RRPs also increased the number of BMCs (Figure 1).

### Effects of RRPs on histopathological appearance of bone marrow

**Figure 2.**
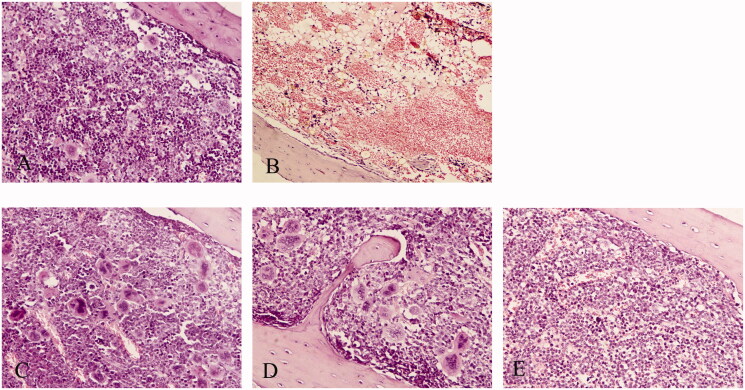
Influence of RRPs on histopathological changes in bone marrow in mice with aplastic anaemia. (A) Bone marrow, normal control (100×); (B) bone marrow, anaemic control (100×); (C) bone marrow, high-dose RRPs (100×); (D) bone marrow, medium-dose RRPs (100×); and (E) bone marrow, low-dose RRPs (100×). Stain, haematoxylin and eosin.

Compared to normal mice, anaemic mice showed decreased bone-marrow hyperplasia, decreased haematopoietic tissue area, decreased haematopoietic cells, increased adipocytes, and decreased megakaryocytes, accompanied with interstitial edoema. At 7 days after the intraperitoneal injection of different doses of RRPs, the haematopoietic cells in the bone marrow gradually increased, and non-haematopoietic cells decreased; interstitial edoema was relieved, and the number of megakaryocytes and the area of haematopoietic tissue increased (Figure 2).

### *In vivo* effects of RRPs on proliferation of bone-marrow haematopoietic progenitors

**Figure 3.**
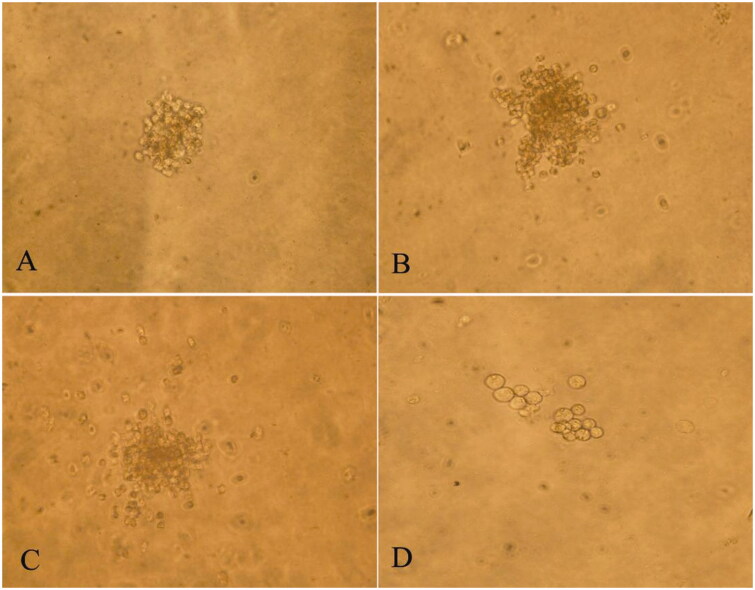
Culture of bone-marrow haematopoietic progenitor cells. (A) CFU-E, (B) BFU-E, (C) CFU-GM, and (D) CFU-Meg. Normal control, unstained (200×).

**Figure 4.**
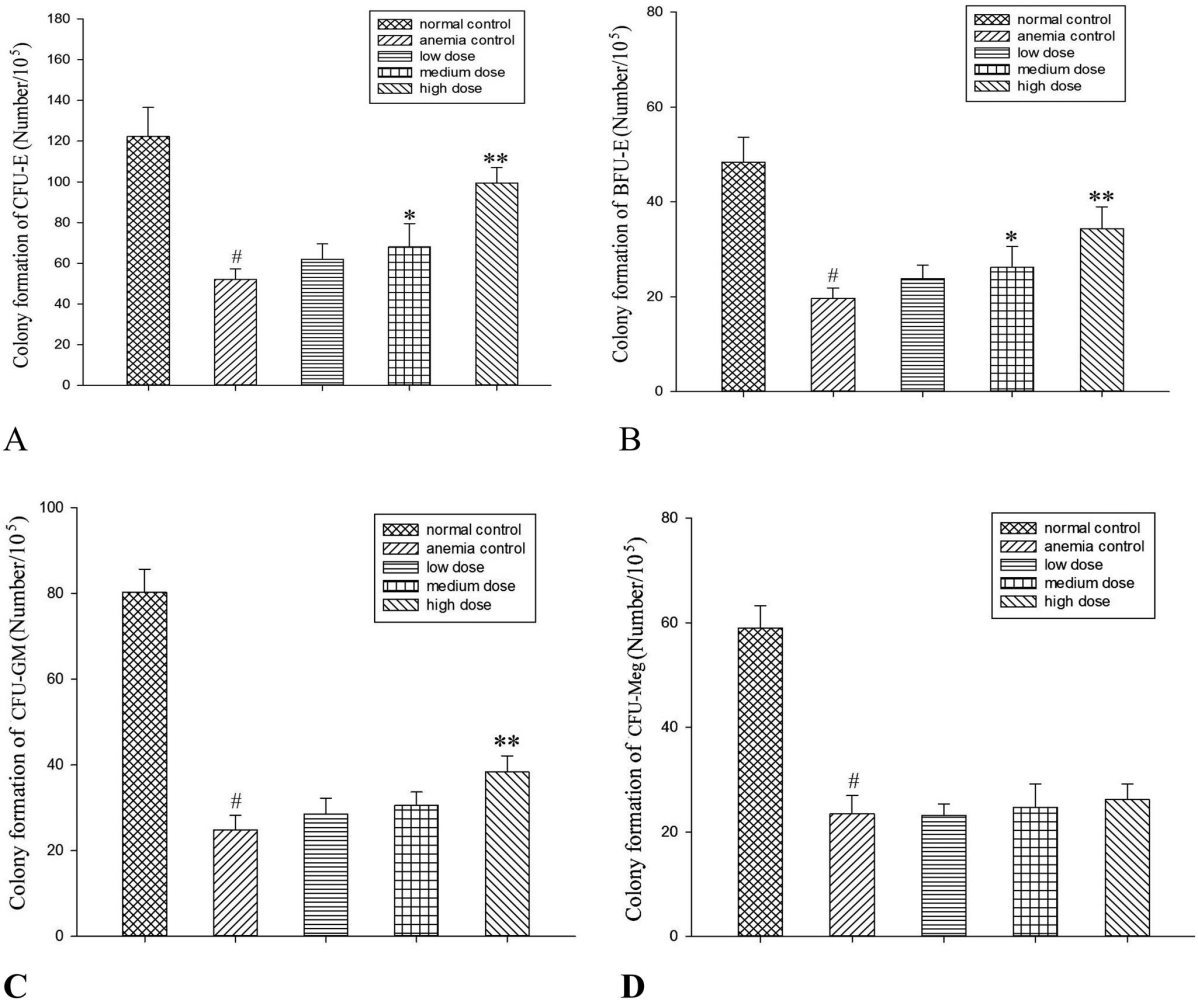
*In vivo* effects of RRPs on proliferation of bone-marrow haematopoietic progenitors. (A) CFU-E, (B) BFU-E, (C) CFU-GM, and (D) CFU-Meg. #*p<*0.01 versus normal controls; *p<0.05 and **p<0.01 versus anaemic controls.

There were more than 50 BFUs-E and CFUs-GM, more than 8 CFUs-E, and more than 3 CFUs-Meg (Figure 3) in the normal control group. Compared to the anaemic control group, the medium-dose RRP group showed significantly increased formation of BFUs-E and CFUs-E (*p* < 0.05), while the high-dose RRP group showed significantly increased formation of CFUs-GM, BFUs-E, and CFUs-E (*p* < 0.01; Figure 4).

### Effects of RRPs on Fas and FasL protein expressions

**Figure 5.**
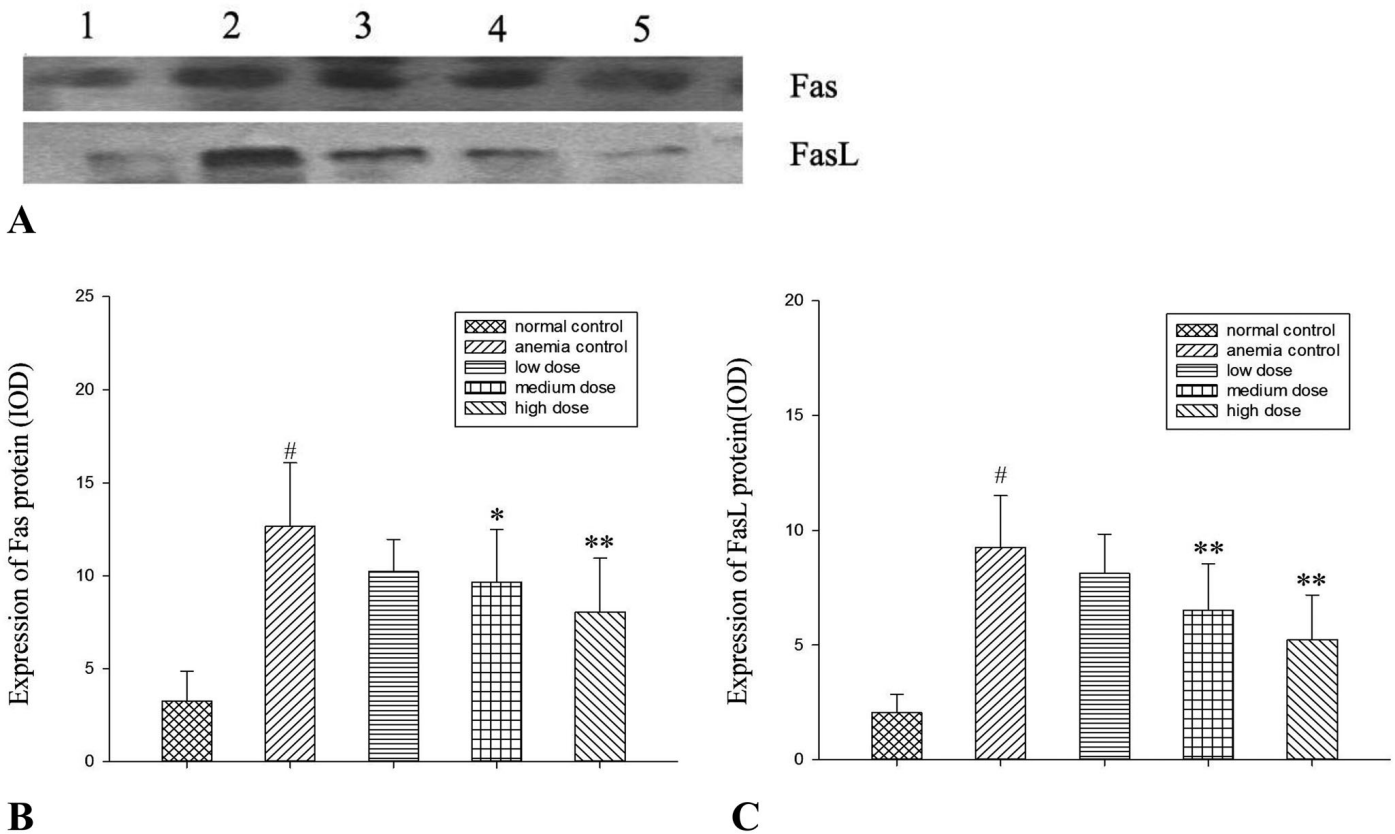
Protein expression of Fas and FasL. (A) Western blot analysis of Fas and FasL. Lane 1, normal controls; lane 2, anaemic controls; lane 3, low-dose RRPs; lane 4, medium-dose RRPs; and lane 5, high-dose RRPs. (B) Integrated optical density (IOD) of Fas. (C) IOD of FasL. #*p<*0.01 versus normal controls; *p<0.05 and **p<0.01 versus anaemic controls.

Western blotting revealed that the protein expression levels of Fas and FasL in BMCs were decreased in all RRP groups, and the decreases were significant in the high-dose group (*p* < 0.01; Figure 5).

### *In vitro* effects of RRPs on proliferation of bone-marrow haematopoietic progenitors

**Figure 6.**
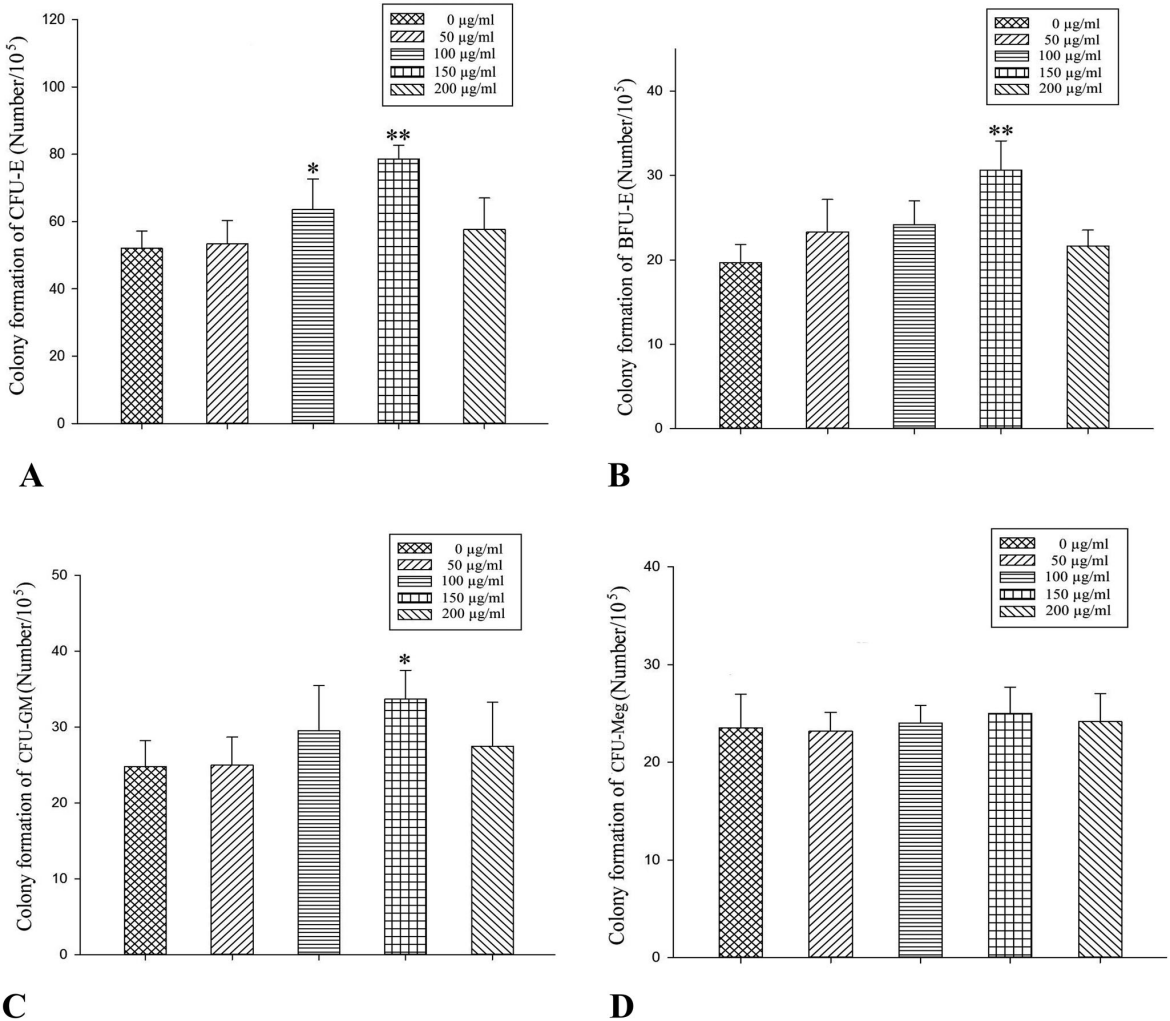
*In vitro* effects of RRPs on proliferation of bone-marrow haematopoietic progenitors. (A) CFU-E, (B) BFU-E, (C) CFU-GM, and (D) CFU-Meg. *p<0.05 versus the 0 µg/mL RRPs group.

The optimal *in vitro* concentration of RRPs for promoting the formation of CFUs-E, BFUs-E, and CFUs-GM was 150 μg/mL. RRPs did not significantly promote the formation of CFUs-Meg (Figure 6).

### Effects of RRPs on BMSC proliferation

**Figure 7.**
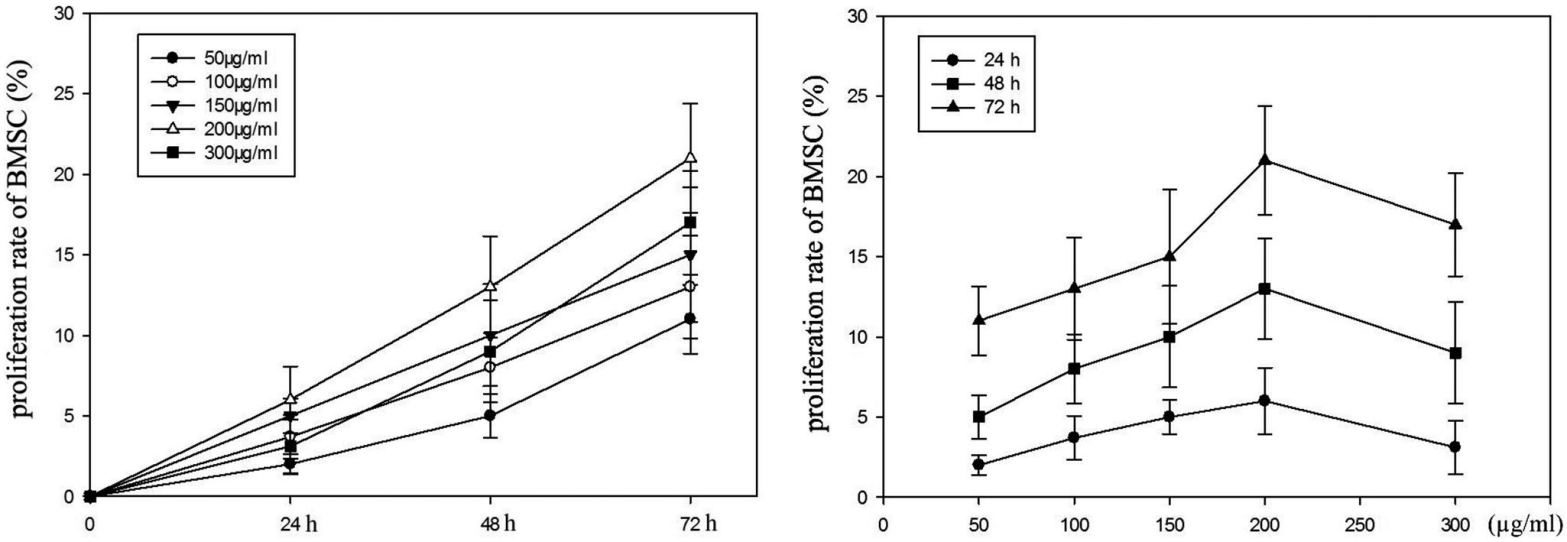
*In vitro* effects of RRPs on BMSC proliferation.

After 48 h of culture, stromal cells in the bone marrow were arranged in a fusiform shape. With the extension of the culture time, the BMSC proliferation rates increased in both the normal and model groups, especially within the concentration range of 150–300 μg/mL (Figure 7).

## Discussion

*Rhodiola rosea* is a rare medicinal plant that grows in cold, high-altitude regions. The main components of *R. rosea* are salidroside, *p-*tyrosol, and polysaccharides. Recent pharmacological research has revealed that these components help promote the plant’s adaptability to environmental stress and regulate its physiological functions. These components also have antihepatotoxic activities and anti-fatigue and anti-ageing functions (Udintsev and Schakhov 1991; Hu et al. 2010; Li et al. 2011; Qian et al. 2011; Lee et al. 2013; Leung et al. 2013). The present research focussed on the influence of RRPs on bone-marrow haematopoietic function in mice with aplastic anaemia. The purpose of this research was to enhance the exploitation and utilization of *R. rosea*.

This experiment induced aplastic anaemia in mice through the combined use of ^60^Coγ irradiation and cyclophosphamide. The model groups showed evident reductions in white blood cells, red blood cells, platelets, and BMCs. In addition, the bone marrow showed a decrease in the haematopoietic tissue area and an increase in adipocytes. These changes indicated that the aplastic anaemia model had been successfully constructed. Seven days after the intraperitoneal injection of different doses of RRPs, the peripheral blood cells of the mice in the model groups recovered to differing degrees. High doses of RRPs had an evident effect on leukocytes, erythrocytes, and haemoglobin. Bone-marrow haematopoietic cells gradually increased, and non-haematopoietic cells decreased. Interstitial edoema was relieved, and the haematopoietic tissue area increased. These findings suggested that RRPs potentially promoted the recovery of peripheral blood cell counts and haematopoietic tissues.

The *in vivo* experiments showed that medium and high doses of RRPs could promote the formation of CFUs-E and BFUs-E; low, medium, and high doses of RRPs all promoted the formation of CFUs-GM, but only the high dose produced a statistically significant effect. RRPs showed no considerable effect on CFUs-Meg. These findings indicate that in mice with myelosuppression, RRPs improved haematopoietic function by promoting the proliferation of erythroid cells and granulocytes. These results were consistent with the changes observed in the peripheral blood cell counts. The *in vitro* experiments indicated that the pro-proliferation effects of RRPs on bone-marrow haematopoietic progenitor cells were lower under *in vitro* conditions than under *in vivo* conditions. The proliferation-promoting effects of RRPs were mainly focussed on erythroid progenitor cells in the *in vitro* experiments. Our research indicated that RRPs had direct and indirect effects on haematopoietic progenitor cells, and that this effect was selective.

BMSCs are important components of the haematopoietic microenvironment. They greatly influence the growth, differentiation, and self-renewal of haematopoietic stem cells, and play a crucial role in haematopoietic regulation (Chu and Berek 2013; Chen et al. 2017, 2020). To reveal the influence of RRPs on stromal cells in the bone marrow, we cultured these cells with different doses of RRPs, and analyzed their proliferation using MTT assays. The results showed that the cell proliferation rates were improved in both the normal and model groups in a time-dependent manner. This suggested that RRPs had a good pro-proliferation effect on BMSCs in mice with myelosuppression. Hence, it is conceivable that RRPs could regulate the haematopoietic microenvironment to enhance the recovery of haematopoietic function in bone marrow damaged by radio- and chemotherapy.

FasL is secreted by natural killer cells and activated T cells, and induces apoptosis of target cells via the death receptor Fas/Apo1/CD95; both FasL and Fas mediate the immunocytotoxic death of harmful cells such as tumour cells and virus-infected cells (Pitti et al. 1998). Studies have shown that Fas/FasL-mediated excessive apoptosis of haematopoietic stem/progenitor cells may be an important mechanism of bone-marrow haematopoietic disorders (Maciejewski et al. 1995a, 1995b; Brazil and Gupta 2002; Chen et al. 2015). Our previous experiments involving the streptavidin–biotin complex immunohistochemical method indicated that RRPs can reduce the apoptosis of BMCs by affecting the Fas/FasL-caspase-3 apoptosis signalling pathway in rats with myelosuppression (Li et al. 2008). In the present experiment, we noticed that 7 days after the intraperitoneal injection of different doses of RRPs, the protein expressions of Fas and FasL were decreased in BMCs, indicating that under the present modelling conditions, RRPs could inhibit BMC apoptosis by influencing the Fas/FasL apoptotic pathway and thereby promote the restoration of haematogenesis in mice with aplastic anaemia.

## Conclusions

The present research found that in mice with aplastic anaemia, RRPs at specific concentrations could promote haematopoietic recovery by inhibiting Fas and FasL, increasing peripheral blood cells, facilitating haematopoietic tissue recovery, and promoting haematopoietic progenitor cell and BMSC proliferation. This study indicated some mechanisms underlying the haematopoietic regulatory effects of RRPs. Our research provides a valuable laboratory basis for deeper research on the clinical application of RRPs.

## Data Availability

The datasets generated and analyzed in the present study are available from the corresponding author upon reasonable request.
